# Activation of NF-kB Pathway by Virus Infection Requires Rb Expression

**DOI:** 10.1371/journal.pone.0006422

**Published:** 2009-07-30

**Authors:** Maria A. Garcia, Pedro Gallego, Michela Campagna, José González-Santamaría, Gloria Martínez, Laura Marcos-Villar, Anxo Vidal, Mariano Esteban, Carmen Rivas

**Affiliations:** 1 Centro Nacional de Biotecnología, CSIC, Campus Universidad Autónoma, Madrid, Spain; 2 Departamento de Fisiología, Fac Medicina, Universidad Santiago de Compostela, Santiago de Compostela, Spain; 3 Departamento de Microbiología II, Fac Farmacia, Universidad Complutense de Madrid, Madrid, Spain; University of Hong Kong, Hong Kong

## Abstract

The retinoblastoma protein Rb is a tumor suppressor involved in cell cycle control, differentiation, and inhibition of oncogenic transformation. Besides these roles, additional functions in the control of immune response have been suggested. In the present study we investigated the consequences of loss of Rb in viral infection. Here we show that virus replication is increased by the absence of Rb, and that Rb is required for the activation of the NF-kB pathway in response to virus infection. These results reveal a novel role for tumor suppressor Rb in viral infection surveillance and further extend the concept of a link between tumor suppressors and antiviral activity.

## Introduction

The Rb family of pocket proteins comprises three members Rb, p107 and p130 that have unique and overlapping functions in cell cycle control, differentiation and inhibition of oncogenic transformation [1]. In addition to its classical role in the control of cell progression, the influence of Rb on immune response was also proposed. In this sense, a significant fraction of genes associated with processes related to immune responses, particularly those induced by pathogens or injuries including cell surface molecules, complement factors and genes involved in interferon (IFN) system, are down-regulated in Rb knockout cells [2], [3], [4], [5]. In addition, transforming viral agents contain oncoproteins that inactivate Rb and strikingly, these tumor cells are more susceptible to virus infection than normal cells. Moreover, activation of Rb by IFN treatment has been reported [6], [7], [8]. All together these results suggest that targeting Rb by viral proteins may serve as an advantage for viral replication.

Nuclear factor-kB (NF-kB) is a critical regulator of the immediate early pathogen response, playing an important role in promoting inflammation and in the regulation of cell proliferation and survival [9], [10]. In most cells NF-kB exists as an inactive cytoplasmic complex, whose predominant form is a heterodimer composed of p50 and RelA/p65 subunits, bound to inhibitory proteins of the IkB family. The inactive NF-kB complex is activated in response to a variety of stimuli, including viral and bacterial infections, exposure to proinflammatory cytokines, mitogens and growth factors, and stress-inducing agents [11], [12], which activate the inhibitory kB kinases (IKK). One of these kinases, IKKβ, phosphorylates IkBα, causing its poly-ubiquitination and subsequent degradation by the 26S proteasome. The release of NF-kB from its inhibitor IkBα, allows its nuclear translocation and transactivation of NF-kB target genes. Since gene expression of many proinflammatory and antiviral cytokines is controlled by this factor, the concept emerged that NF-kB and its upstream regulator IKK are essential components of the innate antiviral immune response to infectious pathogens.

In this report, we have analyzed the role of the tumor suppressor Rb in the control of virus replication. We demonstrate that the absence of Rb, but not of related proteins p107 or p130, increases virus replication and that Rb is involved in the activation of the antiviral NF-kB pathway. These results identify a new function for the tumor suppressor Rb establishing a new link between viral infection and tumor suppression.

## Results and Discussion

Mammalian cells respond to viral infection by producing and secreting type I IFN that triggers the expression or post-translational modification of hundreds of cellular genes some of which are implicated in tumor and viral protective networks [13], [14], [15], [16], [17]. One tumor suppressor activated by IFN is Rb [7], [8], a protein that controls cell cycle or differentiation and that may have a role in modulation of immune functions [2], [3], [4], [5], [18], [19], [20], [21].

**Figure 1:**
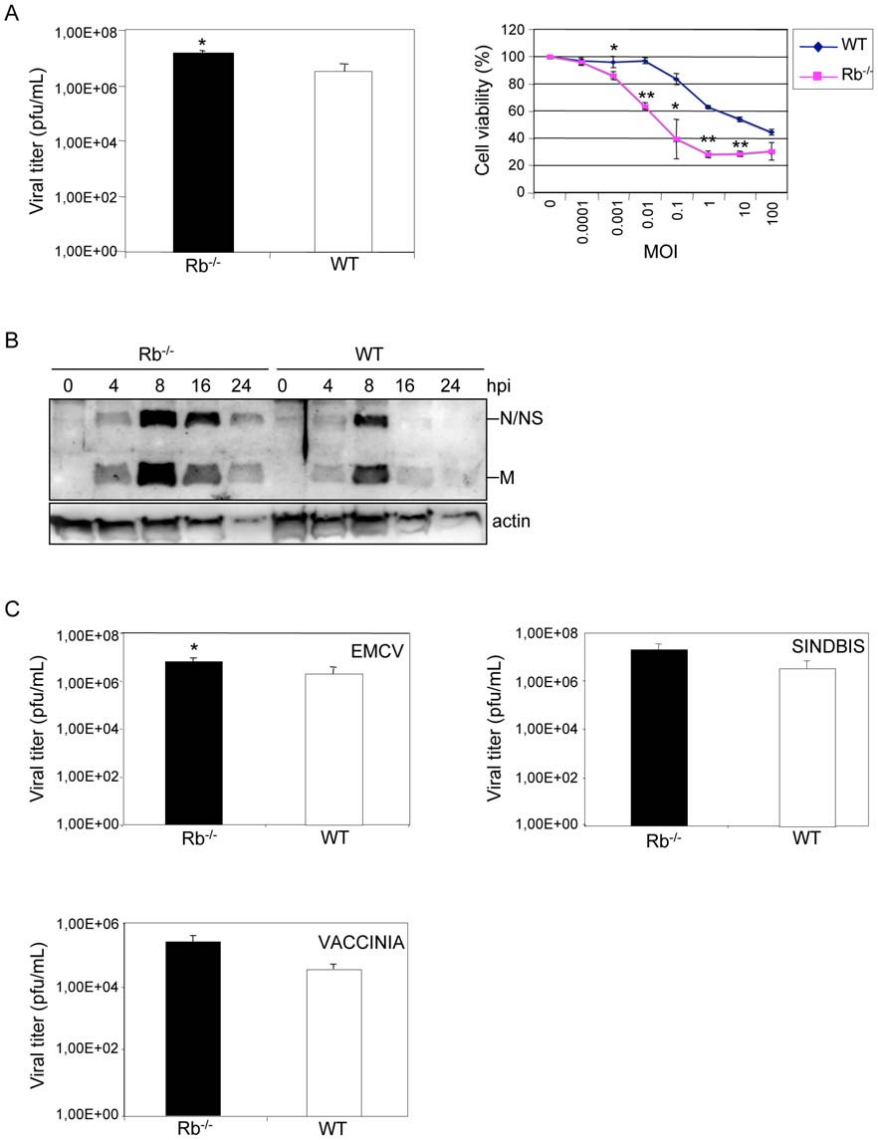
Rb^−/−^ MEFs are more susceptible to virus infection. A, Rb^−/−^ or WT MEFs were infected in duplicate with VSV at M.O.I. of 5 and a quantification of the virus yield after a total destruction of the monolayers was assessed (left panel). The same results were obtained in at least three different experiments and using MEFs derived from three different wild type or transgenic embryos. Data represent means+/−SE for one experiment. *, P<0.05, compared with WT cells, Student's test. Right panel, Rb^−/−^ or WT MEFs were infected with VSV at the indicated M.O.I. and 24 h after infection, cell viability was quantified as described in Methods. Indicated values are means+/−SE of triplicate wells for one experiment. The same results were obtained in at least three different experiments and using MEFs derived from three different wild type or transgenic embryos. *, P<0.05, **, P<0.005, compared with WT cells, Student's test. B, Rb^−/−^ or WT MEFs were infected with VSV at a M.O.I. of 0.5 and at the indicated times, Western-blotting analysis using antibodies against VSV proteins was performed. C, Rb^−/−^ or WT MEFs were infected with EMCV, sindbis virus or vaccinia virus at M.O.I. of 5 and when the viability of all the cells was lost quantification of the intracellular virus yield (vaccinia virus) or the virus present in the supernatant (EMCV or sindbis virus) was measured. Data represents means+/−SE for four experiments. *, P<0.05 compared with WT cells, Student's test.

To study the role of Rb family proteins in the cell's response to virus infections, wild type or triple KO for Rb, p107 and p130 (TKO) MEFs were infected with vesicular stomatitis virus (VSV) at a multiplicity of infection (M.O.I.) of 5 plaque-forming units (PFU) per cell, enough to infect all cells, and the amount of viral progeny was measured by titration of the supernatants from infected cultures after all cells had died as a result of the infection. Viral production from WT cells was around one log lower than from TKO cells (supplementary Figure S1A), indicating that absence of Rb family proteins facilitates viral infection. To test individual requirements, similar experiments were performed in MEFs derived from Rb^−/−^, p107^−/−^, p130^−/−^ or WT mice. Single Rb^−/−^ cells showed around one log higher VSV production than MEFs from littermate WT animals (Figure 1A, left panel). In contrast, no differences were observed between the amount of viral progeny recovered from p130^−/−^, p107^−/−^ or WT cells (supplementary Figure S1B). These data indicate that removing Rb alone from cells increases VSV replication. These results were confirmed through the measurements of the cytopathic effect. Rb^−/−^ or WT cells were infected with VSV at different M.O.I. and 24 h after infection cytopathic effect was evaluated. A clear decrease in cell survival was observed after infection of Rb^−/−^ derived MEFs in comparison with WT cells (Figure 1A, right panel). In contrast, an increase in cytopathic effect was not observed in p107^−/−^or p130^−/−^ compared with WT derived MEFs (supplementary Figure S2). In agreement with these data, the lack of Rb resulted in an increase in viral protein synthesis. MEFs were infected with VSV at a M.O.I. of 0.5 and viral protein synthesis was analyzed at various times after infection. A stronger synthesis of VSV proteins was detected in Rb^−/−^ cells, in comparison with WT MEFs (Figure 1B). All together these data demonstrate that absence of Rb but not p107 or p130 enhances VSV replication. Next, in order to determine if the absence of Rb has also a benefitial effect on the replication of other viruses, we quantified virus progeny after extensive cell killing of WT or Rb^−/−^ MEFs by infection with encephalomyocarditis virus (EMCV; picornavirus RNA positive strand), sindbis virus (similar to VSV, RNA negative strand virus) or vaccinia virus (DNA virus) at M.O.I. of 5. Similar to what was found in VSV infection, the virus yields of sindbis, EMCV or vaccinia virus in WT cells were lower than in Rb^−/−^ cells (Figure 1C), although statistical analysis revealed that only the differences found after EMCV infection were significantly different. These results suggest that the contribution of Rb to the control of virus infection differs depending on the virus.

**Figure 2:**
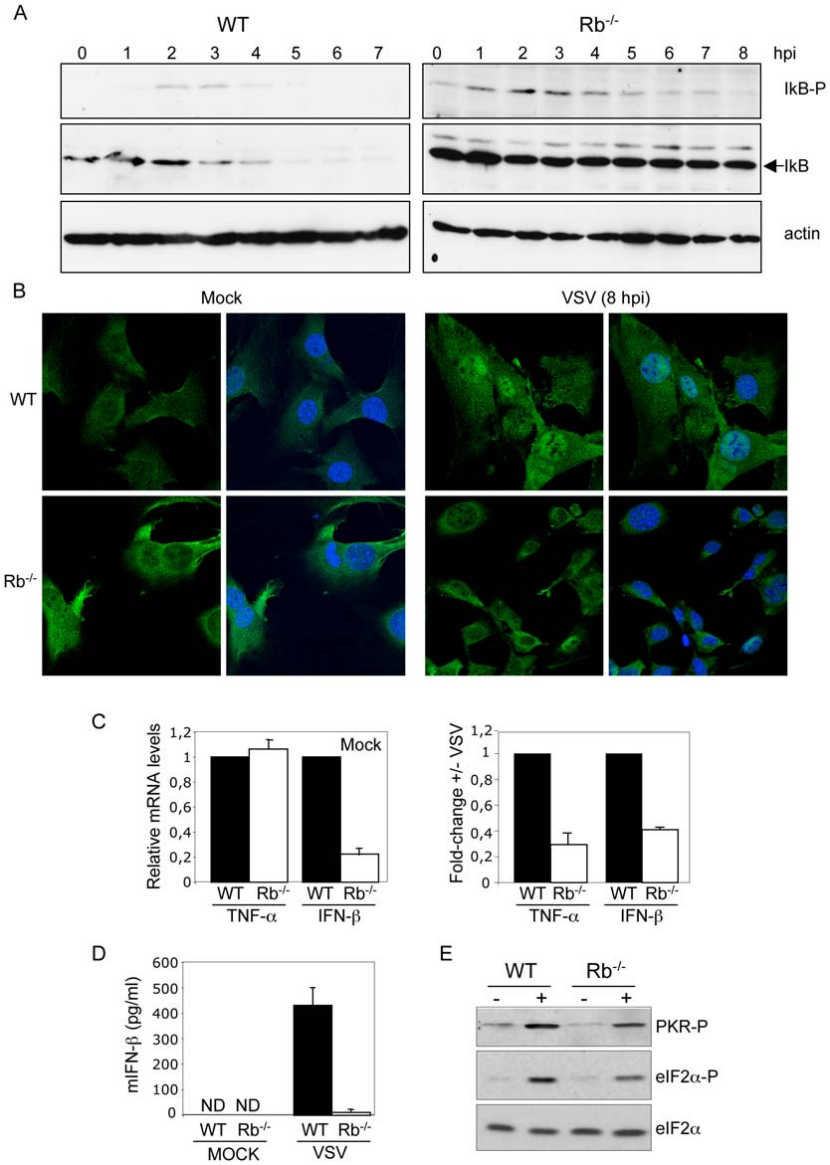
Rb expression is required for NF-kB pathway activation in response to VSV infection. A, Kinetics of IkBα phosphorylation on serine 32 and serine 36 and total IkB upon VSV infection at M.O.I. of 5 PFU/cell in wild type (left panel) or Rb^−/−^ (right panel) primary MEFs were assessed by Western-blotting analysis. B, Wild type or Rb^−/−^ MEFs were infected with VSV at M.O.I. of 5 and 8 h after infection cells were fixed, immunostained for p65 (green) and DAPI (blue). C, Wild type or Rb^−/−^ MEFs were infected with VSV at M.O.I. of 5 and 12 h after infection total RNA was isolated. After reverse transcription the samples were amplified by TaqMan-based QRT-PCR using taqman probes for TNF-α, IFN-β, and GADPDH, and analyzed. The expression levels were determined relative to GAPDH and represented as fold change in Rb^−/−^ cells relative to the expression detected in WT cells. All errors bars indicate mean+/−SE. D, MEFs were infected with VSV at M.O.I. of 5 and 24 h after infection IFN-β production in the cell culture supernatants was measured by ELISA. All errors bars indicate mean+/−SE. ND, not detected. E, Rb^−/−^ or WT MEFs were infected with VSV at M.O.I. of 5 and 7 h after infection, Western-blotting analysis using the indicated antibodies was performed.

VSV infection induces several cellular immunomodulatory pathways with antiviral activity such as the NF-kB pathway. The initial key segment of the NF-kB activation pathway involves IKK activation, IKK phosphorylation of IkBα, and consequent IkBα degradation [22]. Analysis of the activation of this pathway was then carried out in WT or Rb^−/−^ cells. Cells were infected with VSV at M.O.I. of 5 and at different times after infection, IkB phosphorylation and IkB degradation were assessed by Western-blotting. As shown in Figure 2A, IkB phosphorylation was detected by 2 h after VSV infection in WT cells. In agreement with these results IkBα degradation was evident by 3 h after VSV infection of WT cells and was complete by 5 hpi (Figure 2A). In parallel, analysis of Rb^−/−^ cells revealed a basal level of phosphorylated IkB that was slightly increased between 1 and 4 hpi. However, no degradation of IkB was detected at any time after VSV infection of Rb^−/−^ cells, indicating Rb-dependent IkB degradation in response to VSV infection. The second key segment of the NF-kB activation pathway is nuclear translocation of NF-kB subunits and their dimerization and binding to the kB enhancer. Nuclear translocation of NF-kB in response to VSV infection, as a consequence of IkB degradation, was then tested in both WT and Rb^−/−^ MEFs by immunofluorescence. As shown in Figure 2B, nuclear translocation of the p65 subunit of NF-kB was clearly detected at 8 h after infection in WT cells. However, most of the Rb^−/−^ cells showed cytoplasmic staining for p65 at 8 hpi, confirming that VSV-induced nuclear translocation of p65 was also dependent on Rb. The NF-kB transcription factor promotes the expression of many different genes implicated in the antiviral response. To explore the correlation between the nuclear translocation of NF-kB and the induction of NF-kB targets, upregulation of TNF-α or IFN-β genes at 12 h after VSV infection, in WT and Rb^−/−^ MEFs was measured. Semiquantitative RT-PCR showed that relative TNF-α gene transcription levels in non-infected Rb^−/−^ cells was similar to that detected in WT cells (Figure 2C, left panel). Interestingly, a downregulation of the steady-state levels of IFN-β mRNA in Rb^−/−^ cells in comparison with WT cells was found (Figure 2C, left panel), as it has been reported for the interferon-induced Ifi202 gene [23]. Reduced transactivation of both TNF-α and IFN-β in Rb^−/−^ cells in response to VSV infection was observed (Figure 2C, right panel). The impairment of IFN-β production in the absence of Rb after VSV infection was confirmed at the protein level by enzyme-linked immunosorbent assay (ELISA) (Figure 2D). These data indicate that a defect in the activation of the NF-kB pathway and, consequently, in the production of IFN-β in Rb^−/−^ MEFs may explain their increased susceptibility to VSV infection. Activation of NF-kB and eIF2-α by VSV occur in a dsRNA-dependent protein kinase (PKR)-dependent manner [24], [25]. Analysis of PKR activation in Rb^−/−^ and WT MEFs in response to VSV infection revealed decreased levels of both phospho-PKR and phospho-eI2F-α proteins in Rb^−/−^ cells (Figure 2E). Taken together, these results demonstrate the requirement of Rb to activate NF-kB in response to VSV infection and point to PKR as a possible mediator.

**Figure 3:**
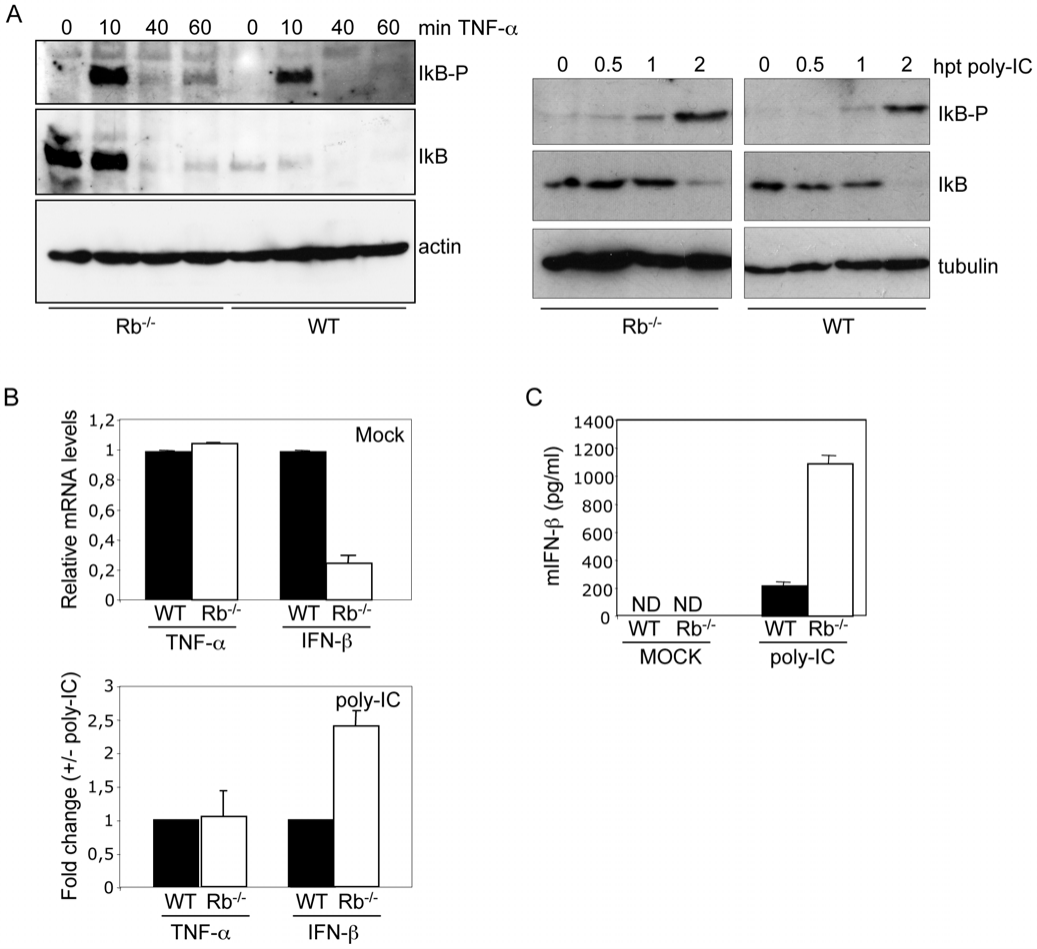
Rb-independent activation of NF-kB in response to TNF-α or poly-IC treatment. A, MEFs derived from WT or Rb^−/−^ mice were treated with 20 ng/ml TNF-α (left panel) or transfected with poly-IC (right panel) and at the indicated times Western-blotting analysis of the indicated proteins was carried out. B, WT or Rb^−/−^ MEFs were transfected with poly-IC and 4 h after transfection total RNA was isolated. After reverse transcription the samples were amplified by TaqMan-based QRT-PCR using taqman probes for TNF-α, IFN-β, and GADPH and analyzed. The expression levels were determined relative to GADPH and represented as fold change in Rb^−/−^ relative to the expression detected in WT cells. C, WT or Rb^−/−^ MEFs were transfected with poly-IC and 8 h after transfection IFN-β production in the cell culture supernatants was measured by ELISA. Error bars indicate mean+/−SE. ND, not detected.

PKR has also a role in NF-kB activation after poly-IC or TNF-α treatment [26]. In order to determine if Rb is required for NF-kB activation in response to these stimuli, both IkB phosphorylation and degradation after TNF-α or poly-IC treatment in WT or Rb^−/−^ cells was assessed. Both treatments induced phosphorylation and degradation of IkB in both WT and Rb^−/−^ MEFs, indicating that Rb is not required for TNF-α or poly-IC-induced IkB activation or degradation (Figure 3A). Poly-IC treatment also induced the activation of the NF-kB targets, IFN-β and TNF-α, as measured by semiquantitative RT-PCR (Figure 3B) and led to an increased IFN-β production in the supernatant, of both WT and Rb^−/−^ MEFs (Figure 3C). Interestingly, IFN-β mRΝΑ and protein production in response to poly-IC was higher in cells lacking Rb. All together these results indicate that Rb is required for NF-kB activation in response to specific stimulus. In this sense, since RIG-1 has been shown to be essential for VSV recognition [27], [28], and MAD-5 is the principal sensor for transfected poly-IC [28], [29], these results suggest a role of Rb downstream of RIG-1 in the pathway leading to IFN response.

Other transcription factor that plays a critical role in type I IFN production in response to viral infection is IRF3. Upon detection of viral components by the cellular sensors, virus-activated kinases phosphorylate IRF3 that in cooperation with NF-kB induce the transcription of type I IFN genes [30]. Analysis of IRF3 activation in WT or Rb^−/−^ MEFs at different times after VSV infection show phosphorylation of IRF3 in both WT and Rb^−/−^ cells (Supplementary Figure S3). This result reinforces the idea that the NF-kB pathway is the mediator of the reduced sensitivity to virus infection observed in the Rb^−/−^ cells.

**Figure 4:**
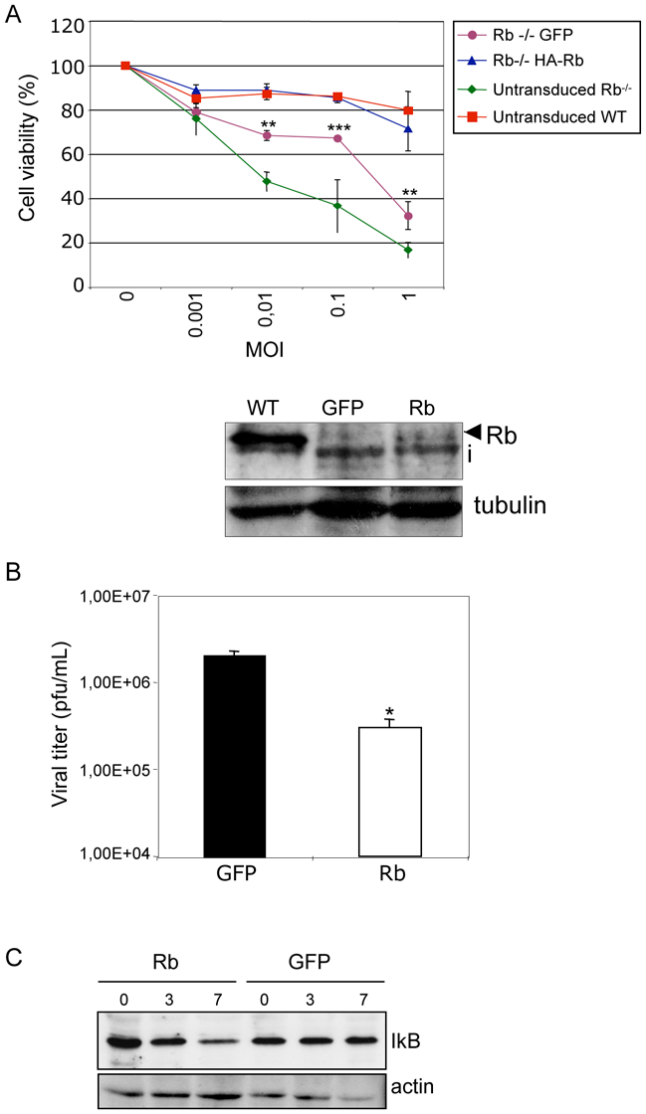
Reconstitution of Rb^−/−^ MEFs increases resistance to VSV infection. A, MEFs WT, Rb^−/−^, and Rb^−/−^ MEFs transduced with retroviral vectors encoding for Rb or GFP were infected with VSV at the indicated M.O.I. After 24 h of infection, cell viability was determined and represented as means+/−SE of triplicate wells (upper panel). **, P<0.005, ***, P<0.0005, Rb-transduced cells compared with GFP-transduced cells, Student's test. Whole cell extracts from Rb WT and transduced Rb^−/−^ cells were immunoblotted with anti-Rb antibody (lower panel). i, inespecific band. B, Reduction in viral yield after Rb reconstitution of Rb^−/−^ MEFs. Virus yield assay of the supernatant of the Rb^−/−^ MEFs transduced with the indicated retroviral vectors after the viability of all the cells was lost as a result of VSV infection (5 M.O.I.). *, P<0.05 Rb-reconstituted cells compared with GFP-transduced Rb^−/−^ cells, Student's test. C, Rb^−/−^ MEFs transduced with the indicated retroviral vectors were infected with VSV at M.O.I. of 5 PFU/cell and at the indicated times after infection, cells were collected, extracts prepared and immunoblot analysis using antibodies against total IkB or actin were performed.

NF-kB activation during viral infection has been interpreted as a protective response of the host to the viral pathogen [31]. Consequently, one would expect that molecules interfering with the NF-kB pathway would have antiviral activity. Thus, we tested the assumption that reconstitution of Rb^−/−^ cells would recover IkB degradation in response to virus infection and would increase control of virus replication. We reintroduced Rb into Rb^−/−^ MEFs by expressing the full-length mouse protein from a retroviral construct and analyzed the development of cytopathic effect. Interestingly, transduction of Rb^−/−^ MEFs with a vector expressing GFP increased the resistance of the cells to be infected by VSV, likely due to the stress involved by three successive rounds of retrovirus transduction. However, expression of Rb increased even higher the resistance of the Rb^−/−^ MEFs to develop cytophatic effect after VSV infection, to similar extent to that observed in the WT cells (Figure 4A). Similarly, lower viral titer from reconstituted cells was obtained (Figure 4B). These data demonstrate that the virus-sensitive phenotype of Rb^−/−^ is directly associated with the absence of Rb. In addition, MEFs transduced with Rb showed some degradation of IkB at 3 h after infection that was increased at 7 h, while no clear degradation was detected in the Rb^−/−^ cells transduced with a vector expressing GFP (Figure 4C). Together these results indicate that Rb has a role in the control of virus infection and that Rb is required for the degradation of IkB in response to virus infection, strongly supporting the NF-kB pathway as the mediator of the antiviral activity induced by Rb.

Although a regulation of NF-kB pathway by Rb in response to TNF-α has been demonstrated, the mechanism is not well defined and seems to be dependent of the cell type, cell context and stimulus [32], [33], [34], [35]. So far, this is the first time it has been demonstrated that Rb is implicated in the control of NF-kB pathway, and specifically in IkB degradation, upon virus infection. In conclusion, our results show that Rb is required for an efficient activation of the NF-kB pathway in response to virus infection, contributing to the control of viral replication and revealing novel features about the Rb function.

## Materials and Methods

### Mice, cell cultures, virus and reagents

p130^−/−^ [36], p107^−/−^ [37] and Rb^+/−^ [38] mice have been previously described. MEFs were isolated from heterozygous matings and cultured as described previously [39]. Generation of triple KO MEFs has been reported [40]. MEFs derived from each KO were assayed in parallel with MEFs derived from the corresponding WT littermates. All MEFs were used before spontaneous immortalization (before passage 6). Green African monkey BSC-40 cells were cultured following a standard procedure. Infections were carried out using VSV of Indiana strain, EMCV, vaccinia virus Western Reserve-strain or sindbis virus and virus yields were measured by plaque assays in BSC-40 cells. A retroviral construct encoding a full length murine Rb [41] was kindly provided by Kostis Alevizopoulos. Retrovirus production and transduction of the target cells were carried out according to methods described previously [42]. Supernatants for ELISA were collected 24 h after VSV infection and 8 h after Poly-IC transfection. Murine IFN-β ELISA kit was acquired from PBL.

### Cell cytolysis induced by VSV

Cells were grown in 96-well plates to 100% confluence and then were infected with different VSV M.O.I. At 24 h after infection, cytolysis was determined by crystal violet staining as previously described [43]. The percentage of viable cells was calculated assuming the survival rate of uninfected cells to be 100%.

### Immunoblotting

Confluent monolayers of cells were infected at M.O.I. of 0.5 or 5 PFU/cell as indicated and cells were lysed directly into SDS sample buffer at different times after infection. Extracts were separated by SDS-PAGE, transferred to nitrocellulose, and incubated with the following antibodies: anti-IkB-α (Cell Signaling Technology), -phospho ser32 and ser36 IkB-α (Cell Signaling Technology), -actin (MP Biomedicals), -phospho PKR (Calbiochem), -eIF2α (Santa Cruz Biotechnology) or –phospho-eIF2α (Biosource International).

### RNA analysis

MEFs were seeded onto 100-mm-diameter dishes and infected with VSV at M.O.I. of 5 PFU/cell. Twelve hours after infection, cells were recovered and total RNA was extracted using the RNeasy mini kit (Qiagen) and reverse transcription (RT-PCR) was performed using the reverse transcription system kit (Promega). Real-time semiquantitative PCR was performed using an ABI7700 instrument and Taqman system (Applied Biosystems). Taqman probes recognizing TNF-α (Mm99999068-m1), IFN-β (Mm00439552-s1) and GADPH (Mm99999915-g1) were used.

### Immunofluorescence and confocal microscopy

MEFs were seeded onto glass coverslips and infected with VSV at M.O.I. of 5 PFU/cell. At 8 h after infection, cells were fixed and stained as described previously [44]. Antibody against p65 (Santa Cruz) was used, followed by Alexa 488-conjugated anti-rabbit immunoglobulin (Molecular Probes). Analysis of the samples was carried out with a Bio-Rad Radiance 2100 confocal laser microscope and images were stored and processed with Laser Pix software package (Bio-Rad Laboratories).

## Supporting Information

Figure S1 (0.19 MB TIF)

Figure S2 (0.27 MB TIF)

Figure S3 (0.21 MB TIF)
