# Supplementary figures and images for: Activation of NF-kB Pathway by Virus Infection Requires Rb Expression
Source: PLoS One. 2009 Jul 30;4(7):e6422. doi: 10.1371/journal.pone.0006422 (PMC2713421; doi:10.1371/journal.pone.0006422)

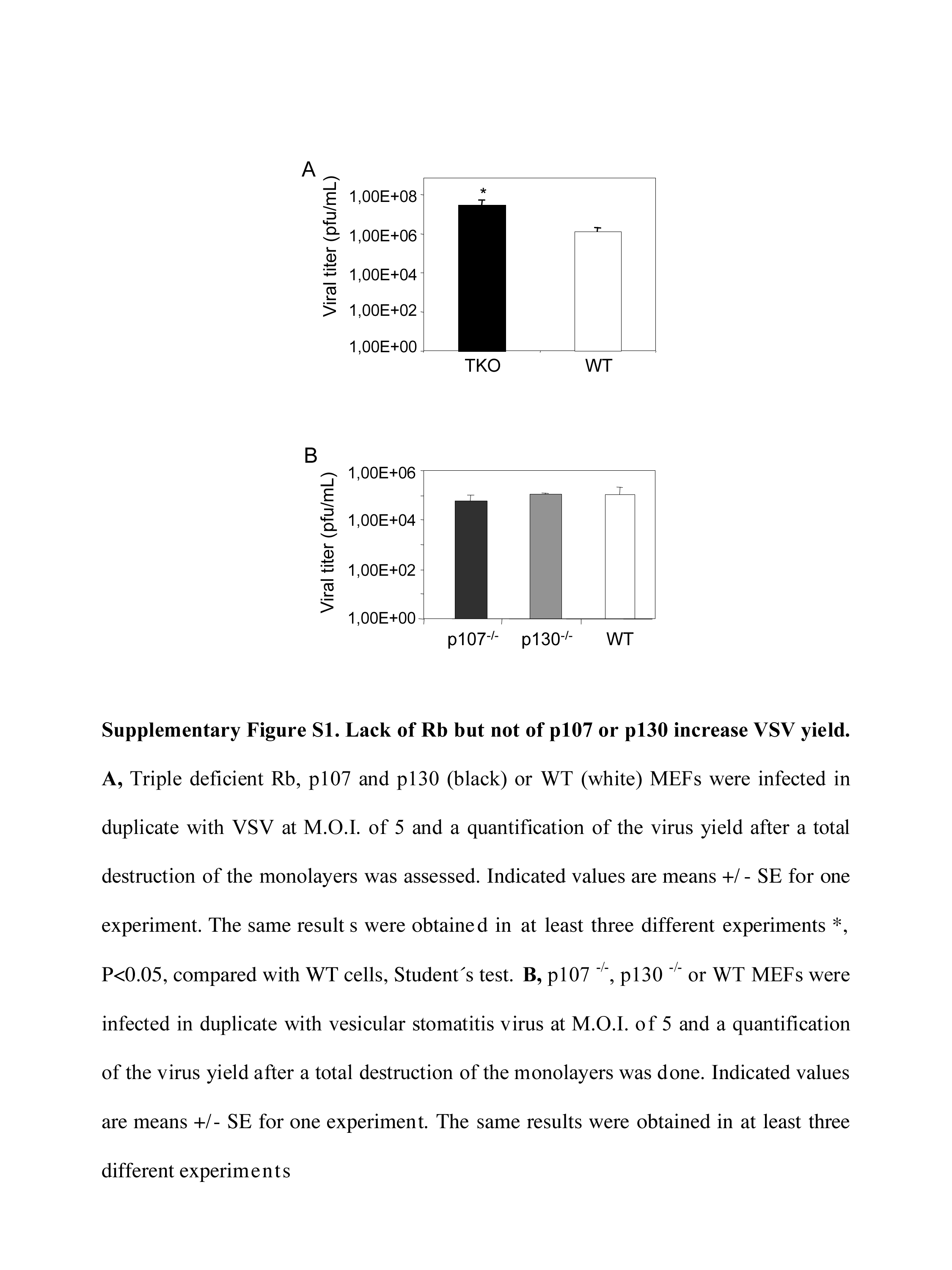

Supplement: Figure S1 — (0.19 MB TIF) [file pone.0006422.s001.tif]

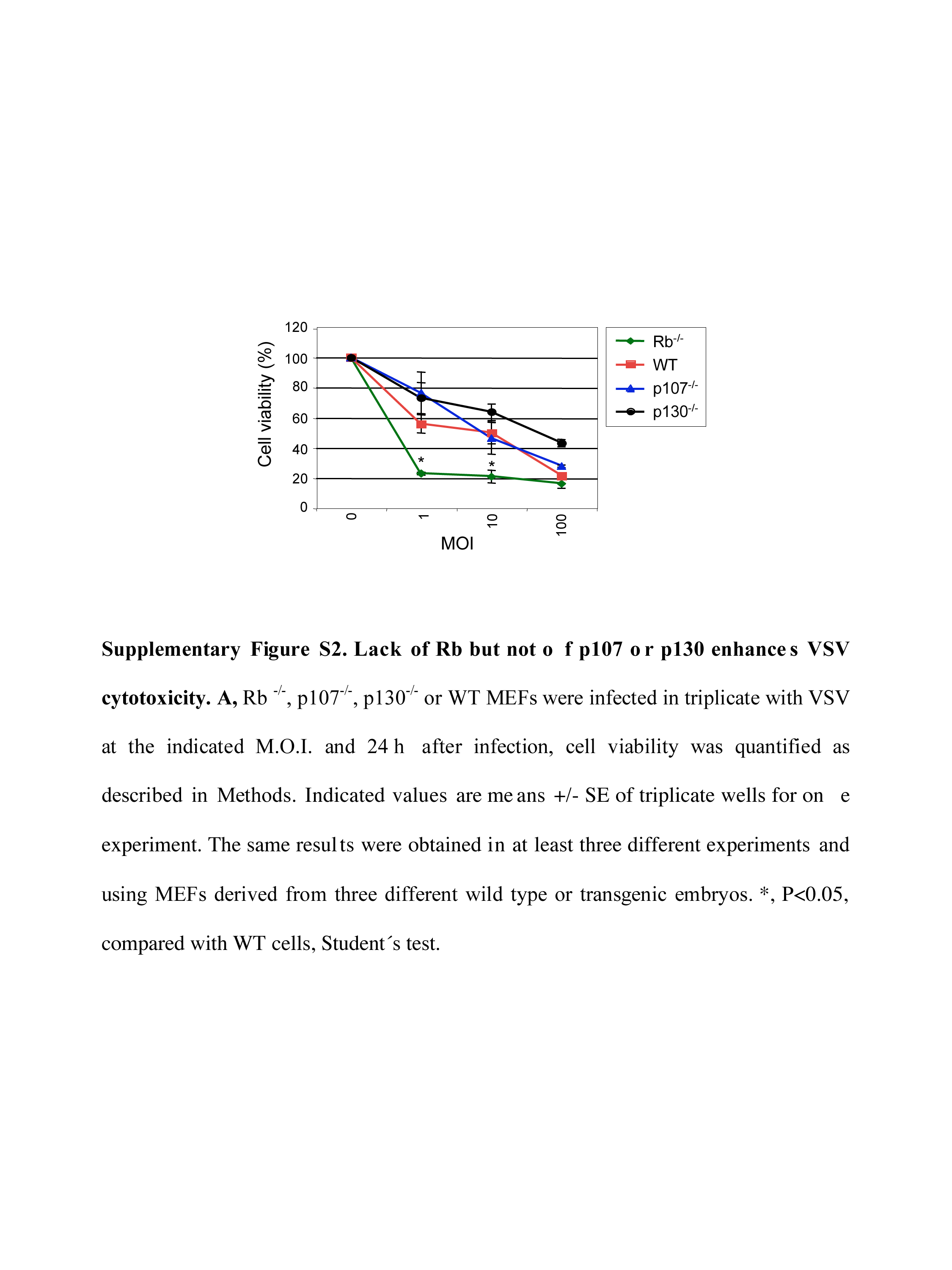

Supplement: Figure S2 — (0.27 MB TIF) [file pone.0006422.s002.tif]

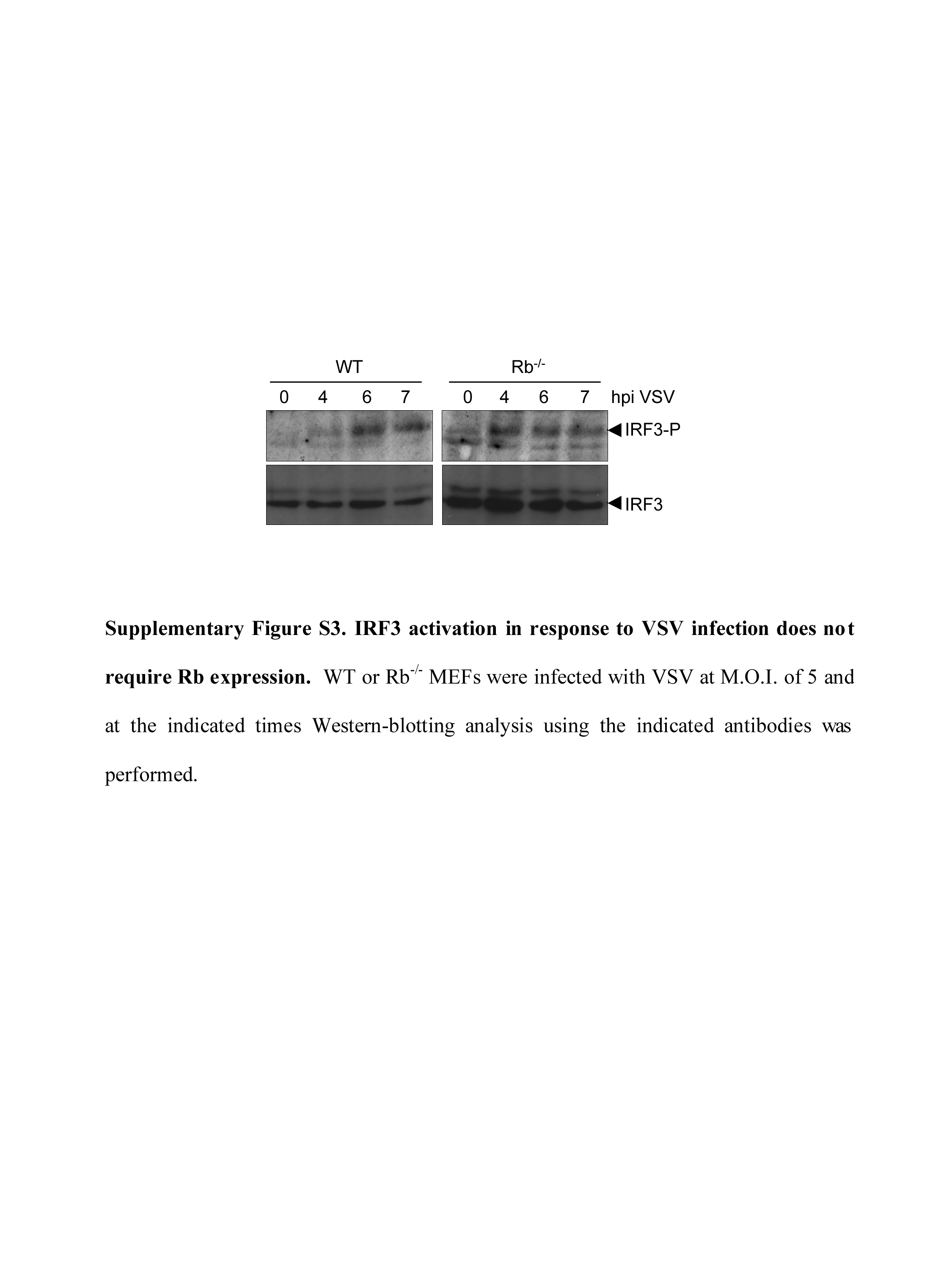

Supplement: Figure S3 — (0.21 MB TIF) [file pone.0006422.s003.tif]
